# Orbital Volumetry in Graves' Orbitopathy: Muscle and Fat Involvement in relation to Dysthyroid Optic Neuropathy

**DOI:** 10.1155/2014/435276

**Published:** 2014-04-02

**Authors:** Moug Al-Bakri, Åse Krogh Rasmussen, Carsten Thomsen, Peter Bjerre Toft

**Affiliations:** ^1^Department of Ophthalmology, Glostrup Hospital, University of Copenhagen, Glostrup, Denmark; ^2^Eye Clinic 2061, Rigshospitalet, University of Copenhagen, Blegdamsvej 9, Copenhagen, Denmark; ^3^Department of Endocrinology, Rigshospitalet, University of Copenhagen, Blegdamsvej 9, Copenhagen, Denmark; ^4^Department of Radiology, Rigshospitalet, University of Copenhagen, Blegdamsvej 9, Copenhagen, Denmark

## Abstract

*Purpose*. We wanted to investigate the relative significance of fat and muscle enlargement in the development of dysthyroid optic neuropathy (DON) in Graves' orbitopathy (GO). *Methods*. Preoperative coronal CT scans of 13 patients with and without DON who subsequently underwent orbital decompression were retrospectively analyzed. Thirteen patients imaged for unilateral orbital fractures served as controls. *Results*. The retrobulbar muscle volume was 2.1 ± 0.5 cm^3^ (mean ± SD) in controls, 4.3 ± 1.5 cm^3^ in GO without DON, and 4.7 ± 1.7 cm^3^ in GO with DON. The retrobulbar fat volume was 5.4 ± 1.6 cm^3^ in controls, 8.7 ± 8.0 cm^3^ in GO without DON, and 9.4 ± 3.1 cm^3^ in GO with DON. The muscle and fat volumes were higher in patients with GO than in controls (*P* < 0.001), but the volumes in orbits with and without DON were not significantly different. The volume of the optic nerve were similar in the 3 groups. The number of apical, coronal 2 mm thick slices with no fat was 2.9 ± 0.9 in normal orbits, it was 4.1 ± 1.0 in GO orbits without DON and 5.3 ± 0.8 in GO orbits with DON (*P* = 0.007). *Conclusion*. Apical muscle enlargement may be more important than orbital fat enlargement in the development of DON. However, the fact that apical crowding and muscle enlargement also occur in orbits without DON suggests that other factors also play a role in the development of DON.

## 1. Introduction

Graves' disease is an autoimmune disease manifesting in the thyroid, in the dermis, and in orbital connective tissues and muscles [1]. Some 20–50% of Graves' patients develop symptomatic Graves' orbitopathy (GO) [2].

The time association between GO and thyroid disease varies; GO can occur before, during, or after the onset of hyperthyroidism and, less frequently, in euthyroid or hypothyroid cases. GO can occur as unilateral or bilateral disease [3].

The diagnosis of GO is made clinically assisted by biochemical thyroid evaluation and often magnetic resonance (MRI) or computed tomography (CT). The symptoms and signs of active GO include eye discomfort and watering eyes, double vision, eyelid retraction, proptosis, eyelid edema, strabismus, chemosis, disfigurement, corneal problems, and dysthyroid optic neuropathy (DON) [2, 4]. The latter is the most serious manifestation of GO. It is therefore of special interest to identify disease elements associated with the development of DON. DON occurs in 3.4–8% of patients and is thought to be due to optic nerve compression in the orbital apex by enlarged muscles [3–5]. Cigarette smoking, male gender, restrictive strabismus, and rapidly progressive disease are known to predispose to the development of DON [6]. The presence of DON can be established by using simple clinical criteria such as reduced visual acuity and the finding of impaired colour vision by using Ishihara colour plates [4].

Computed tomography and magnetic resonance imaging are both performed in the management of GO. Both axial and coronal images are useful [4]. CT has proven to be excellent in identifying orbital pathology and in visualizing the degree of extraocular muscle and orbital fat enlargement [7–9], and it is often the preferred imaging technique for investigating DON [5]. Some patients with GO have enlarged muscles (muscle GO), some have increased orbital fat (fat GO), and others have both enlarged muscles and increased orbital fat [4].

The present study was undertaken to investigate the presence of muscle and fat enlargement in patients with Graves' disease complicated with GO with or without DON. We also looked at optic nerve volume, as it may be decreased as a result of compression from fat and muscle tissue in orbits with DON.

## 2. Patients and Methods

### 2.1. Patients without GO

Thirteen consecutive patients (6 women, age range 41–77 years, median age 65 years; and 7 men, age range 26–89, median age 34 years) who were seen in the eye clinic and imaged for unilateral orbital fractures were used as a control group. The orbit contralateral to the trauma was used.

### 2.2. Patients with GO

Twenty-one orbits of 13 consecutive patients (8 with bilateral GO and 5 with unilateral GO) who had undergone orbital decompression in the years 2008–2012 and who had preoperative coronal CT scans available were included. DON was present in seven of the included orbits and not in 14 of the orbits. Eight patients had bilateral GO and 5 unilateral GO. There were 11 women (age range 27–81 years, median age 48) and 2 men (age range 58–59 years, median age 58.5).

### 2.3. Clinical Data

Orbital decompression was done because of DON in 7 orbits and for cosmetic rehabilitation in the chronic phase of the disease in 14 orbits. An eye was considered to have DON if the visual acuity decreased by one line or more on the Snellen chart during GO or if the colour vision estimated on the Ishihara plates became affected. The preoperative visual acuity ranged from 6/9 to 6/24 in eyes with DON. The colour vision was affected in all orbits with DON. The preoperative visual acuity in eyes without DON ranged from 6/6 to 6/9 in all but one eye (with a visual acuity of 9/18 because of cataract), and the colour vision was normal in all eyes. All 13 patients were euthyroid preoperatively.

The time from the very first eye symptom(s) as reported by the patient until the operation was performed was 6–44 months in patients with DON and 11–111 months in patients without DON.

At some time point during the disease, all 5 patients (7 orbits) with DON received intravenous methylprednisolone and 2 GO patients without DON had oral prednisolone.

Hertel measurements ranged from 21 to 27 mm in eyes with DON and from 20 to 29 mm in eyes without DON (*P* = 0.3).

### 2.4. Methods

All the patients included in the study had their orbital CT scans reviewed on a personal computer using WEB1000 software (Kodak Eastman Company). The areas of the muscles and the optic nerve and the entire orbital volume behind the globe were obtained by drawing a region of interest along the margins of each individual structure in every image slice behind the globe. The area of each structure was multiplied by the image thickness, 2 mm, and the contributions from each slice were added together. The fat volume was calculated by subtracting the volumes of the optic nerve and the muscles from the total orbital volume behind the globe. In this study, the orbital connective tissue and blood vessels were included in orbital fat measurements. The superior oblique muscles were difficult to identify and the contributions from these muscles were included in the rectus muscle measurements. Images from the orbital apex where the muscles could not be separated from the optic nerve were not included in the calculations.

Since changes in the window and centre settings resulted in slightly different measurements on the CT images, all images were measured with the same settings: 40 HU and 300 HU, respectively.

### 2.5. Apical Orbital Crowding

Muscles and the optic nerve are frequently indistinct in the orbital apex. The number of apical, coronal 2 mm thick slices without any fat was used to objectively quantify orbital apical crowding (see Figure 3). The higher the number, the greater the apical crowding.

### 2.6. Statistical Method

Statistical analyses were performed using SPSS software version 19. Student's 2-tailed *t*-test for unpaired data was used for statistical comparisons between groups. Any *P* value < 0.05 was considered statistically significant.

## 3. Results

### 3.1. Patients without GO

We evaluated images of 13 orbits from 13 patients with contralateral orbital trauma. Comparison of normal female and male orbits showed that male orbits had 14% greater orbital muscle volume and female orbits had 10% more orbital fat volume than male orbits. However, this was not statistically significant (*P* = 0.2 and *P* = 0.3).

### 3.2. Patients with GO

**Figure 1:**
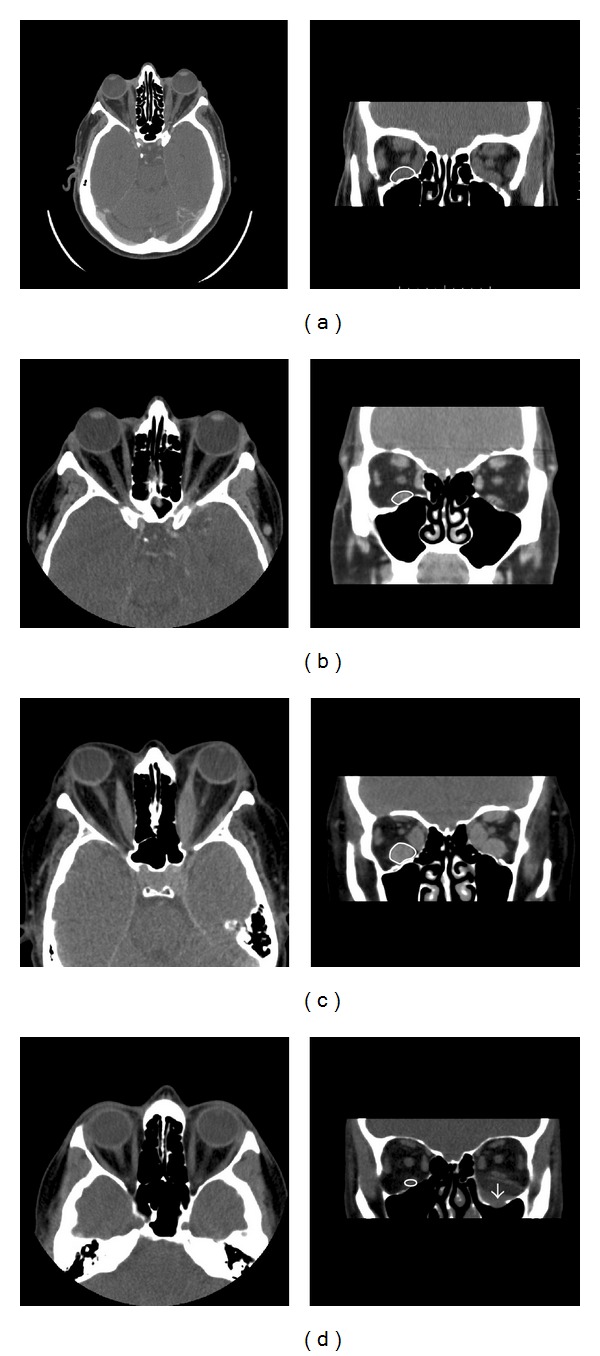
Axial and coronal CT images of 3 representative patients with Graves' disease complicated with GO ((a)–(c)) and one control patient (d). The regions of interest are drawn on each coronal slice. Patient (a) has GO with predominant muscle enlargement. Patient (b) has GO with predominant enlargement of orbital fat in the right orbit whereas the left orbit is less affected. Patient (c) has enlargement of both orbital muscles and fat in both orbits and the visual acuity was reduced to 6/18 in the left orbit as a result of DON. The visual acuity was reduced to 6/6 in the right orbit. The patient shown in (d) had a floor fracture in the left orbit and the right orbit served as one of the 13 normal controls.

**Figure 2:**
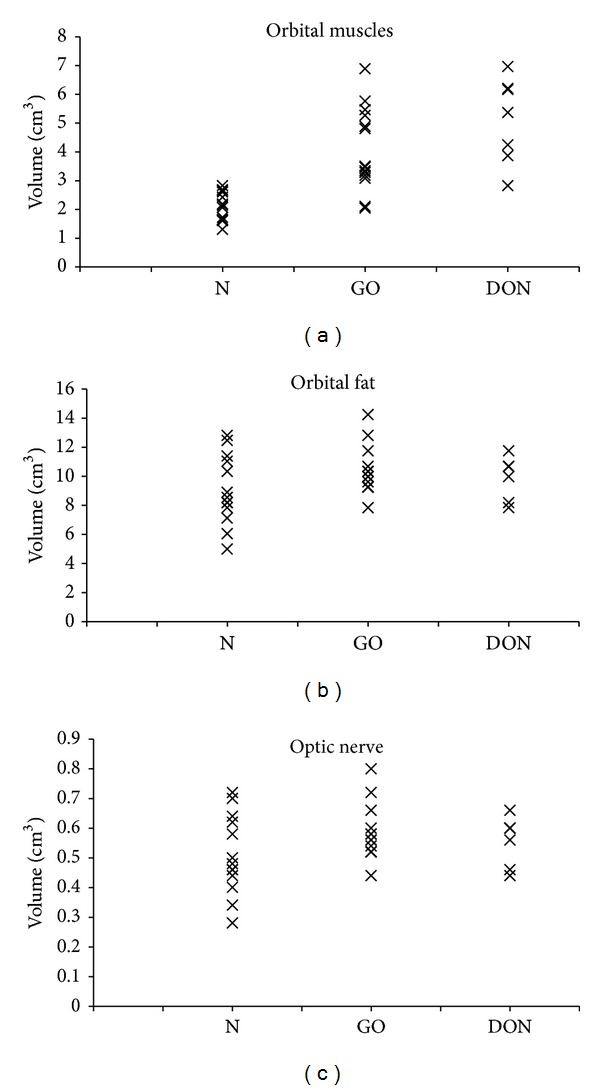
(a) The volumes of muscles in normal orbits, GO orbits without DON, and GO orbits with DON. The mean muscle volume was higher in GO patients than in controls (*P* < 0.001), but the mean volumes in orbits with and without DON were not significantly different. Note that the muscle volume was over the normal range in all 7 orbits with DON, whereas this was only the case in 11 of 14 orbits without DON. (b) The volumes of orbital fat in normal orbits, GO orbits without DON, and GO orbits with DON. The mean fat volume was higher in GO patients than in controls (*P* < 0.001), but there was no statistically significant difference between orbits with and without DON. (c) The mean volumes of the optic nerve in normal orbits, GO orbits with DON, and GO orbits without DON. No significant difference between the 3 groups was observed. N = normal orbits, GO = GO orbits without DON, and DON = GO orbits with DON. (b) The volumes of orbital fat in normal orbits, GO orbits without DON and GO orbits with DON. The mean fat volume was higher in GO patients than in controls (*P* < 0.001), but there was no statistically significant difference between orbits with and without DON. (c) The mean volumes of the optic nerve in normal orbits, GO orbits with DON, and GO orbits without DON. No significant difference between the 3 groups was observed. N = normal orbits, GO = GO orbits without DON, and DON = GO orbits with DON.

**Figure 3:**
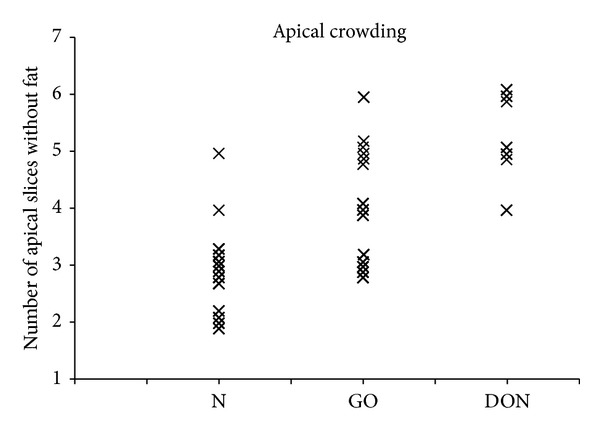
The number of apical, coronal 2 mm thick slices without any fat was used to quantify apical crowding. The apical crowding was higher in GO patients than in the controls (*P* < 0.001), and it was higher in orbits with DON than in orbits without DON (*P* = 0.007). N = normal orbits, GO = GO orbits without DON, and DON = GO orbits with DON.

Images of 21 orbits from 13 patients were evaluated. Representative examples of coronal and axial CT scans of 4 different patients are shown in Figure 1. The measurements of muscle, fat, and the optic nerve volumes are shown in Figures 2(a), 2(b), and 2(c), respectively. The relative amount of muscle and fat varies in each of the 3 groups: normal orbits and GO orbits with and without DON, Figures 2(a) and 2(b).

The retrobulbar muscle volume was 2.1 ± 0.5 cm^3^ (mean ± SD) in normal controls, 4.3 ± 1.5 cm^3^ in GO patients without DON, and 4.7 ± 1.7 cm^3^ in GO patients with DON. The mean muscle volume was higher in patients with GO than in controls (*P* < 0.001), but the mean extraocular muscle volumes in orbits with and without DON were not significantly different (*P* = 0.6). However, the muscle volume was over the normal range in all 7 orbits with DON, whereas this was only the case in 11 of 14 orbits without DON.

The percentage of the total retrobulbar orbital volume that was muscle was calculated to be 26.7% ± 3.2 (mean ± SD) in normal orbits, 31.4% ± 5.4 in GO orbits without DON, and 31.8% ± 1.6 in GO orbits with DON. The percentage of muscle was higher in patients with GO than in the controls (*P* < 0.001), but the mean percentages of muscle in orbits with and without DON were not significantly different (*P* = 0.8).

The mean retrobulbar fat volume was 5.4 ± 1.6 cm^3^ in normal controls, 8.7 ± 8.0 cm^3^ in GO patients without DON, and 9.4 ± 3.1 cm^3^ in GO patients with DON. The mean fat volume was higher in patients with GO than in the controls (*P* < 0.001), but the mean fat volumes in orbits with and without DON were not significantly different (*P* = 0.7). The retrobulbar fat was within the normal range in 4 of the orbits with DON.

The mean volume of the optic nerve was 0.5 ± 0.1 cm^3^ in normal controls, 0.6 ± 0.1 in GO patients without DON, and 0.6 ± 0.04 cm^3^ in GO patients with DON. The difference between groups was not statistically significant.

### 3.3. Apical Crowding

The mean number of apical, coronal 2 mm thick slices without any fat was 2.9 ± 0.9 in normal orbits, 4.1 ± 1.0 in GO orbits without DON, and 5.3 ± 0.8 in GO orbits with DON. This measure of apical crowding was significantly higher in orbits with DON than in orbits without DON (*P* = 0.007).

### 3.4. Sources of Error

The measurements on the CT images were performed by the same investigator. The reproducibility of the method was analyzed by repeating the measurements in 4 orbits after a two-month time interval. In each case of muscle and fat measurement, the second measurement was within 16% (mean 5%) of the initial measurement. However, the optic nerve measurement was less accurate and the second measurement differed by up to 32% (mean 21%).

The muscle volume of the patients with predominantly muscle disease may have been underestimated because crowding in the apex occurs in these patients especially. Crowding makes it impossible to distinguish muscle from optic nerve. According to the study protocol, the volume contribution from the most apical slices is not added to the total volume. This possible error results in a small underestimation of muscle volume in patients with the largest muscles.

The number of 2 mm thick apical coronal slices is also affected by motion artefacts, which were more frequent in patients with orbital trauma.

## 4. Discussion

In this study, we examined the orbital volumes of retrobulbar muscle, fat, and optic nerve in orbits from patients with Graves' disease complicated with GO and compared them with normal orbits. In 7 of the 21 orbits, vision was affected because of optic neuropathy, and an interesting question is whether any of the orbital volumes are associated with the presence of DON.

### 4.1. Muscle Enlargement and DON

As expected, the muscle volume was significantly higher in patients with GO than in the controls, but—somewhat surprisingly—the mean muscle volumes in orbits with and without DON did not differ significantly. Also, the percentage of the total retrobulbar orbital volume that was muscle was not significantly different. Thus, it appears that muscle enlargement per se does not result in DON.

Barrett et al. [10] suggested a method capable of quantifying impingement of the muscle on the optic nerve and therefore a useful indicator of the risk of DON. The horizontal Barrett's index is calculated as the percentage of orbital width occupied by lateral and medial muscles. The vertical Barrett's index is calculated as the percentage of orbital height occupied by superior and inferior muscles. The largest value is considered to be Barret's index [10]. Because Barrett's index is measured halfway between the globe and the apical orbit, it is likely that this method is less sensitive in detecting orbits with predominantly apical muscle enlargement. However, Barret et al. did find a higher Barret's index in 31 patients with DON than in patients without DON. The Barrett's index values (mean ± SD) were 62.67% ± 8.14 and 49.44% ± 10.94 in the groups with and without DON, respectively (*P* < 0.001) [10]. Although the study by Barret et al. suggested that extraocular muscle enlargement is the most important diagnostic feature and indicator of the severity of GO, cases of DON without muscle enlargement have been reported. Anderson et al. presented atypical cases of DON with normal-sized or minimally enlarged muscles. They concluded that these cases demonstrate that muscle enlargement alone is an inadequate basis for diagnosis and visual prognosis [11].

### 4.2. Apical Crowding and DON

A statistically significant difference between apical crowding in normal orbits and GO orbits was observed. Furthermore, there was a significant difference in apical crowding in orbits with and without DON. Our finding of a difference in apical orbital crowding in orbits with and without DON is in agreement with the findings of Chan et al. [12], who suggested that the apical crowding score correlated strongly with clinical DON [5, 12]. However, considering the large overlap of orbital crowding in orbits with and without DON, it appears that apical crowding is not the only factor responsible for the development of DON.

### 4.3. Fat Increase and DON

GO usually presents with muscle enlargement and/or increased orbital fat [13, 14]. However, some authors have demonstrated the presence of a subset of patients with GO who only have increased fat volume but normal muscle volume [15, 16], and our findings confirm this. We observed a statistically significant difference between fat volumes in normal orbits and GO orbits, but the orbital fat volumes varied widely in patients with GO. The fat volumes among GO patients were even within the normal range in 4 of the orbits with DON, and the retrobulbar orbital fat volume was similar in orbits with and without DON. However, our finding of no statistically significant difference in fat volume in orbits without and with DON is in agreement with the results of Goncalves et al. [5], who found a significant difference in apical muscle crowding in orbits with and without DON but no difference regarding orbital fat in the 2 groups. It may be that, in most patients with DON, muscle enlargement is of more importance than fat enlargement in the pathogenesis of DON.

### 4.4. Optic Nerve Stretching and DON

The increase in orbital volume behind the globe will result in optic nerve stretching. The volume of the nerve remains unchanged and the nerve therefore becomes thinner or may take a less curved path in GO. Stretching of the nerve does not appear to play a major role in the development of DON. This is in agreement with the work of Chan et al., who also found similar optic nerve volumes in orbits with and without DON [12].

Another interesting question is why the volumes of fat and muscle are so differently affected in different patients. This has also been described by others, for example, by Regensburg et al. who described 4 subtypes of GO among 95 orbits of GO patients; 25 orbits showed no increase of fat or muscle volume (group 1), 5 orbits only showed fat volume increase (group 2), 58 orbits only showed muscle volume increase (group 3), and 8 orbits showed both fat and muscle volume increase (group 4) [12]. The swelling of muscles and orbital fat/connective tissue is due to inflammatory edema and accumulation of glycosaminoglycans. Infiltrating immunosorbent cells drive the tissue reactivity and orbital fibroblasts secrete cytokines and glycosaminoglycans. These are very hydrophilic components that accumulate in orbital tissues of GO [10]. However, the reasons for the differential enlargement of orbital fat and muscle compartments are incompletely understood. Some of the possibilities could be as follows.

*Different Cytokine Profiles in Muscles and Fat Tissues*. Under the influence of particular cytokines, a group of Thy-1-negative orbital fibroblasts called preadipocytes, present in orbital fat but not in muscles, differentiates into adipocytes. This process of adipogenesis is associated with an increased expression of TSHR and contributes in later stages of GO to further expansion of orbital fat resulting in increased fat volume. The Th1-like cytokines predominate in muscles [10]. 

*Smoking*. Regensburg et al. included 95 untreated GO patients and grouped them according to muscle/fat volume enlargement and their smoking behavior. They suggested that smoking is related to larger extraocular muscle volume in GO patients and that smoking is not related to orbital fat volume. However, this finding was not statistically significant [11]. 

*GO Duration*. Wiersinga et al. suggest that fat volumes were larger in patients with GO duration longer than 1 year compared with duration less than 1 year. They concluded that adipogenesis is a rather late phenomenon in the pathogenesis of GO [10, 11].

## 5. Conclusion

Total retrobulbar muscle and fat volumes do not differ significantly in orbits with and without DON; however, apical crowding appears to be more pronounced in DON. Thus, factors other than muscle enlargement may also be important for the development of DON. These factors could be inflammation-related and/or impaired circulation. Imaging can confirm apical crowding in DON and help select those patients who may benefit from decompression surgery.

## References

[1] Paik JS, Cho WK, Oh EH, Lee SB, Yang SW (2012). Palmitate induced secretion of IL-6 and MCP-1 in orbital fibroblasts derived from patients with thyroid-associated ophthalmopathy. *Molecular Vision*.

[2] Goncalves AC, Gebrim EM, Monteiro ML (2012). Imaging studies for diagnosing Graves’ orbitopathy and dysthyroid optic neuropathy. *Clinics*.

[3] Goncalves AC, Silva LN, Gebrim EM, Matayoshi S, Monteiro ML (2012). Predicting dysthyroid optic neuropathy using computed tomography volumetric analyses of orbital structures. *Clinics*.

[4] McKeag D, Lane C, Lazarus JH (2007). Clinical features of dysthyroid optic neuropathy: a European Group on Graves’ Orbitopathy (EUGOGO) survey. *British Journal of Ophthalmology*.

[5] Goncalves AC, Silva LN, Gebrim EM, Monteiro ML (2012). Quantification of orbital apex crowding for screening of dysthyroid optic neuropathy using multidetector CT. *American Journal of Neuroradiology*.

[6] Lee JH, Lee SY, Yoon JS (2010). Risk factors associated with the severity of thyroid-associated orbitopathy in Korean patients. *Korean Journal of Ophthalmology*.

[7] Forbes G, Gehring DG, Gorman CA (1985). Volume measurements of normal orbital structures by computed tomographic analysis. *American Journal of Roentgenology*.

[8] Peyster RG, Ginsberg F, Silber JH, Adler LP (1986). Exophthalmos caused by excessive fat: CT volumetric analysis and differential diagnosis. *American Journal of Roentgenology*.

[9] Weis E, Heran MK, Jhamb A (2012). Quantitative computed tomographic predictors of compressive optic neuropathy in patients with thyroid orbitopathy: a volumetric analysis. *Ophthalmology*.

[10] Barrett L, Glatt HJ, Burde RM, Gado MH (1988). Optic nerve dysfunction in thyroid eye disease: CT. *Radiology*.

[11] Anderson RL, Tweeten JP, Patrinely JR, Garland PE, Thiese SM (1989). Dysthyroid optic neuropathy without extraocular muscle involvement. *Ophthalmic Surgery*.

[12] Chan L-L, Tan H-E, Fook-Chong S, Teo T-H, Lim L-H, Seah L-L (2009). Graves ophthalmopathy: the bony orbit in optic neuropathy, its apical angular capacity, and impact on prediction of risk. *American Journal of Neuroradiology*.

[13] Weber AL, Dallow RL, Sabates NR (1996). Graves’ disease of the orbit. *Neuroimaging Clinics of North America*.

[14] Fang ZJ, Zhang JY, He WM (2013). CT features of exophthalmos in Chinese subjects with thyroid-associated ophthalmopathy. *International Journal of Ophthalmology*.

[15] Forbes G, Gorman CA, Brennan MD (1986). Ophthalmopathy of Graves’ disease: computerized volume measurements of the orbital fat and muscle. *American Journal of Neuroradiology*.

[16] Trokel S, Kazim M, Moore S (1993). Orbital fat removal: decompression for Graves orbitopathy. *Ophthalmology*.

